# Brain targeting of 9c,11t-Conjugated Linoleic Acid, a natural calpain inhibitor, preserves memory and reduces Aβ and P25 accumulation in 5XFAD mice

**DOI:** 10.1038/s41598-019-54971-9

**Published:** 2019-12-05

**Authors:** Orli Binyamin, Keren Nitzan, Kati Frid, Yael Ungar, Hanna Rosenmann, Ruth Gabizon

**Affiliations:** 1 Department of Neurology, The Agnes Ginges Center for Human Neurogenetics, Hadassah-Hebrew University Medical Center, Jerusalem, Israel; 2 Chemistry laboratory, Milouda & Migal Laboratories, Merieux Nutrisciences, Milu’ot South Industrial Zone, Akko, Israel

**Keywords:** Cell death in the nervous system, Neurodegenerative diseases

## Abstract

Deregulation of Cyclin-dependent kinase 5 (CDK5) by binding to the activated calpain product p25, is associated with the onset of neurodegenerative diseases, such as Alzheimer’s disease (AD). Conjugated Linoleic Acid (CLA), a calpain inhibitor, is a metabolite of Punicic Acid (PA), the main component of Pomegranate seed oil (PSO). We have shown recently that long-term administration of Nano-PSO, a nanodroplet formulation of PSO, delays mitochondrial damage and disease advance in a mouse model of genetic Creutzfeldt Jacob disease (CJD). In this project, we first demonstrated that treatment of mice with Nano-PSO, but not with natural PSO, results in the accumulation of CLA in their brains. Next, we tested the cognitive, biochemical and pathological effects of long-term administration of Nano-PSO to 5XFAD mice, modeling for Alzheimer’s disease. We show that Nano-PSO treatment prevented age-related cognitive deterioration and mitochondrial oxidative damage in 5XFAD mice. Also, brains of the Nano-PSO treated mice presented reduced accumulation of Aβ and of p25, a calpain product, and increased expression of COX IV-1, a key mitochondrial enzyme. We conclude that administration of Nano-PSO results in the brain targeting of CLA, and suggest that this treatment may prevent/delay the onset of neurodegenerative diseases, such as AD and CJD.

## Introduction

Several pathological features characterize late onset neurodegenerative diseases, such as Alzheimer’s (AD), Parkinson (PD) Creutzfeldt Jacob disease (CJD) diseases and others. One is the accumulation over time and disease advance of aberrantly folded key disease proteins^1^. Another one is the oxidation of such proteins and lipids around it, which may be linked to the impairment of mitochondrial activity and of the proteasomal and lysosomal pathways^2–4^. In genetic cases, linked to mutations in the genes of the designated key disease proteins, accumulation of the aberrant peptides results from the spontaneous misfolding or the mutant proteins^5,6^. Oxidative stress and aberrant aggregation of proteins may also be caused by the truncation of the CDK5 activator p35 to p25 by Calpain^7,8^. CDK5 is an indispensable enzyme for brain development during embryogenesis and subsequently performs important tasks in the adult brain, including higher cognitive functions such as learning and memory formation^9^. Following its binding to p25 as opposed to p35, Cdk5 activity becomes deregulated in several neurological disorders, such as AD, PD and Huntington’s disease, eventually leading to neurotoxicity^10,11^. Pharmacological modulation resulting in inhibition of calpain activity is now considered an important target in the search of treatments for neurodegenerative diseases^12^.

We have shown recently that long-term administration of Nano-PSO (Granagard^TM^), a nanodroplet formulation of Pomegranate Seed Oil comprising large levels of Punicic Acid (PA), delayed disease advance significantly in TgMHu2ME199K mice, modeling for genetic CJD linked to the E200K PrP mutation^13^. Treatment of these genetic mice with Nano-PSO reduced the levels of ROS and restored normal mitochondrial activity and levels of regulatory oxidation factors in brains^14,15^. Interestingly, it has been shown previously that administration of PSO, comprising 80–90% Punicic Acid, as well as an oil produced from Trichosanthes kirilowii^16^, comprising 40% Punicic Acid, to rats or mice, resulted in the rapid metabolism of PA into 9c, 11t Conjugated Linoleic Acid (CLA), while only traces of CLA and no PA were found in brains^17,18^. It has been speculated that the liver could be the main location of the enzymes for the conversion of PA to CLA^17–19^. Concomitantly, CLA was found to be a μ-calpain-specific inhibitor, demonstrating neuroprotective effects following treatment of SH-SY5Y cells with H_2_O_2_ and Aβ_1–42_^20^. CLA also inhibited Aβ oligomerization/fibrillation and decreased the levels of p25 accumulation and tau phosphorylation in cells^20^.

In this project, we first investigated whether administration of Nano-PSO, as opposed to PSO, may target CLA to the brains of the treated mice. Concomitantly, we studied the effect of long-term administration of Nano-PSO to 5XFAD mice, a double transgenic mice line that co-express five familial AD mutations^21^. These mice accumulate Aβ in an age dependent manner and from 4–5 months of age onward present significant memory loss as well as cognitive decline^21^. 5XFAD and other similar mouse lines are widely used to study AD pathogenesis and concurrently test the activity of agents that may treat or prevent the onset of AD^22,23^. In this work, Nano-PSO treated and untreated 5XFAD or WT mice were assessed for their cognitive status, and subsequently their brains tested for the levels of pathological and biochemical markers such as Aβ, COX IV-1 and p25^24^.

We show here that while no PA and only traces of CLA were found in the brains of mice treated with natural PSO, substantial levels of this lipid molecule were detected in the brains of mice treated constantly with Nano-PSO. Next, we demonstrate that long-term administration of Nano-PSO (from weaning-age to 10 months of age) to 5XFAD mice, significantly prevented their age dependent memory impairment, as established by several cognition tests^25,26^. In addition, we present evidence that the brains of Nano-PSO treated 5XFAD mice accumulate reduced levels of both Aβ and p25, indicating this reagent indeed performs as a brain targeted calpain inhibitor^27^. It remains to be established whether Nano-PSO can function as an effective neuroprotector in humans at risk to develop neurodegenerative conditions.

## Results

### Brain accumulation of CLA in mice treated with Nano-PSO

**Figure 1:**
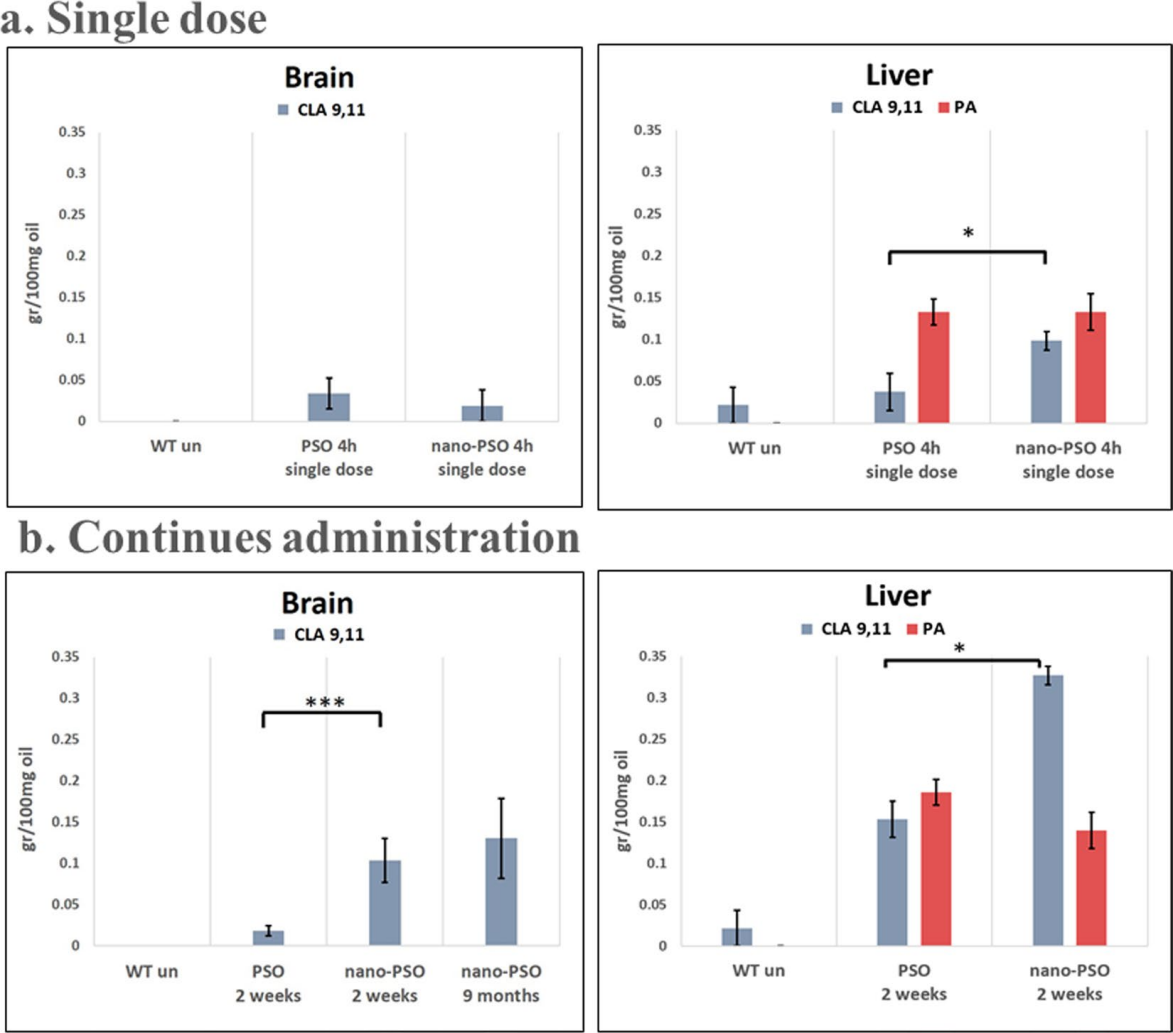
Brain targeting of CLA following administration of Nano-PSO to WT mice. C57B mice were treated either with PSO or with Nano-PSO as described in the methods and subsequently lipids were extracted from brains and livers for detection of PA or CLA. (**a**) PA and CLA after a single dose of PSO or Nano-PSO administration (**b**) PA and CLA after continuous administration of PSO and Nano-PSO. Unpaired t-test: *p < 0.05; ***p < 0.001.

PSO or Nano-PSO were administrated to C57BL/6 mice in several modes. In the first one, 3 months old mice were given a single dose and sacrificed 4 h later, when their livers and brains were collected and frozen for subsequent lipid extraction and analysis (Fig. 1a). In a second mode, PSO or Nano-PSO were administered to the designated mice for two consecutive weeks before brains and livers were collected and analyzed (Fig. 1b). In a third mode, Nano-PSO was administered continuously for 9 months in the drinking water of the mice, and subsequently their brains were analyzed for PA and CLA. Figure 1 shows that, in accordance with published results^17–19^, no PA and only traces of CLA were detected in the brains of mice treated with PSO, either after 4 h or after 2 weeks of administration. Contrarily, significant CLA levels were detected in the brains of mice treated continuously with Nano-PSO. Our results also show that the conversion of PA to CLA in the liver increased significantly when PSO was administrated to the mice as a nano formulation. These results suggest that the increased neuroprotective activity of Nano-PSO, as compared to PSO, may relate both to the rapid metabolism of PA to CLA in the liver, in addition to a more efficient targeting of CLA into the brain. Figure 1 also shows that mice treated for 9 months accumulate similar levels of CLA in their brains as mice treated for 2 weeks, indicating that continues administration of Nano-PSO can maintain this CLA levels indefinitely without causing any side effects, concomitantly with the clinical and pathological beneficial effects shown in the TgMHu2ME199K model^14,28^.

### Nano-PSO prevents cognitive decline of 5XFAD mice

**Figure 2:**
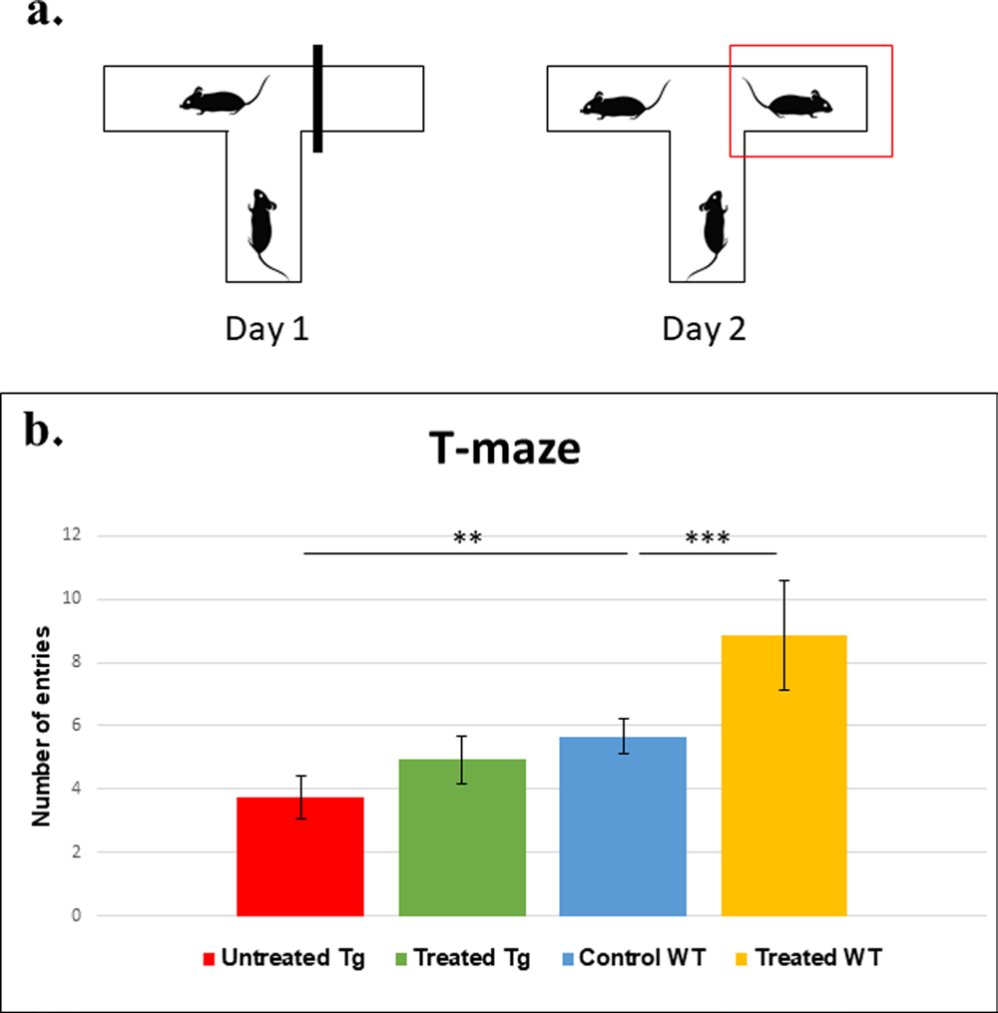
Nano-PSO administration to both 5XFAD and WT mice improved their performance on the T-maze test. 5XFAD and WT mice treated with or without Nano-PSO were subjected to the T-Maze test at 7 months of age as described in the methods. (**a**) cartoon of the experiment (**b**) Number of entries to the new arm on day 2. Statistical analysis were performed by planned contrast test of numbers of entries to unfamiliar arm reveal significant difference between the groups [f(3,38) = 14.009, p < 0.001)].

**Figure 3:**
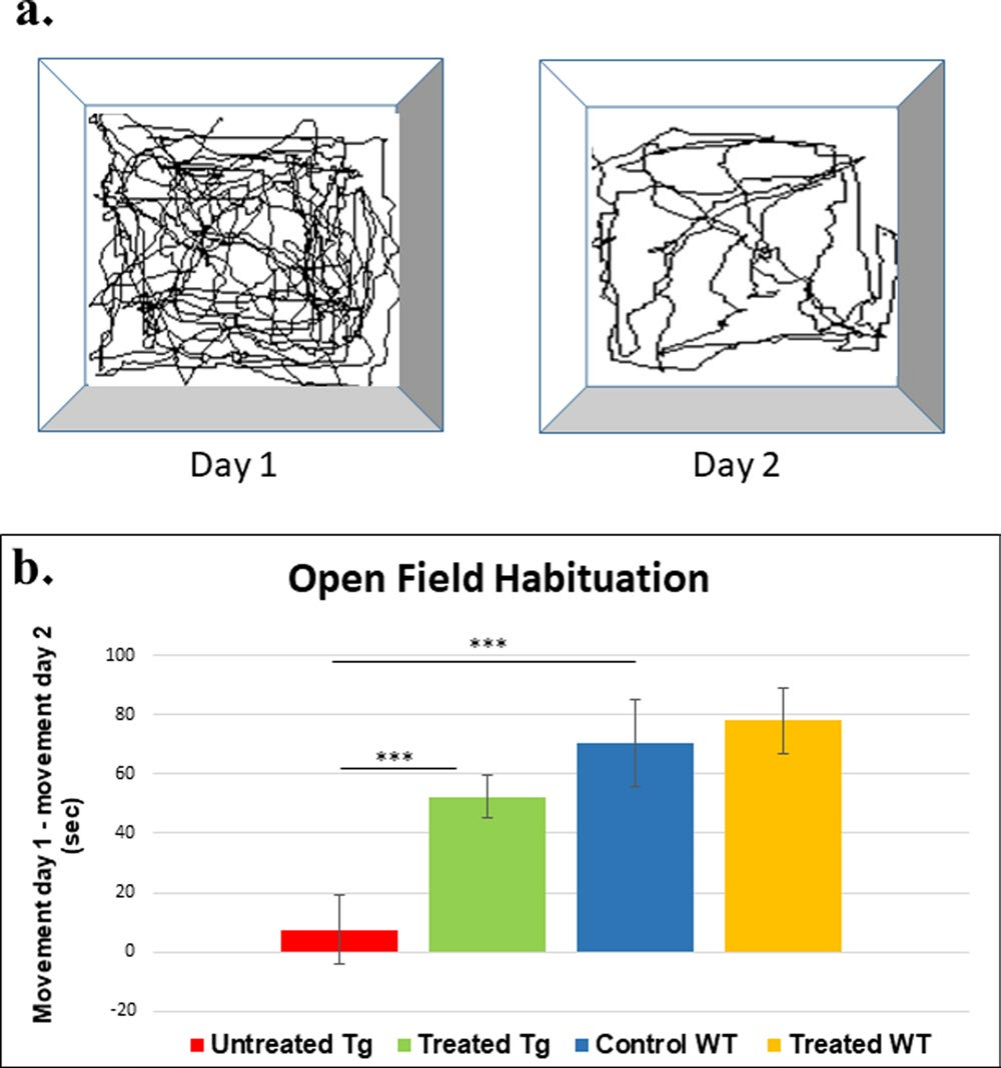
Effect of Nano-PSO treatment on long-term non-associative spatial memory in the open field habituation task. 5XFAD and WT mice treated with or without Nano-PSO were subjected to the open field habituation test at 7 months of age as described in the methods. (**a**) Cartoon of the experiment (**b**) Difference in movement duration between day 1 and day 2. One-way Anova (Tukey post hoc analysis) present significant difference between Tg untreated group (n = 12) and Tg treated group (***p < *0.001*; n = 18).

**Figure 4:**
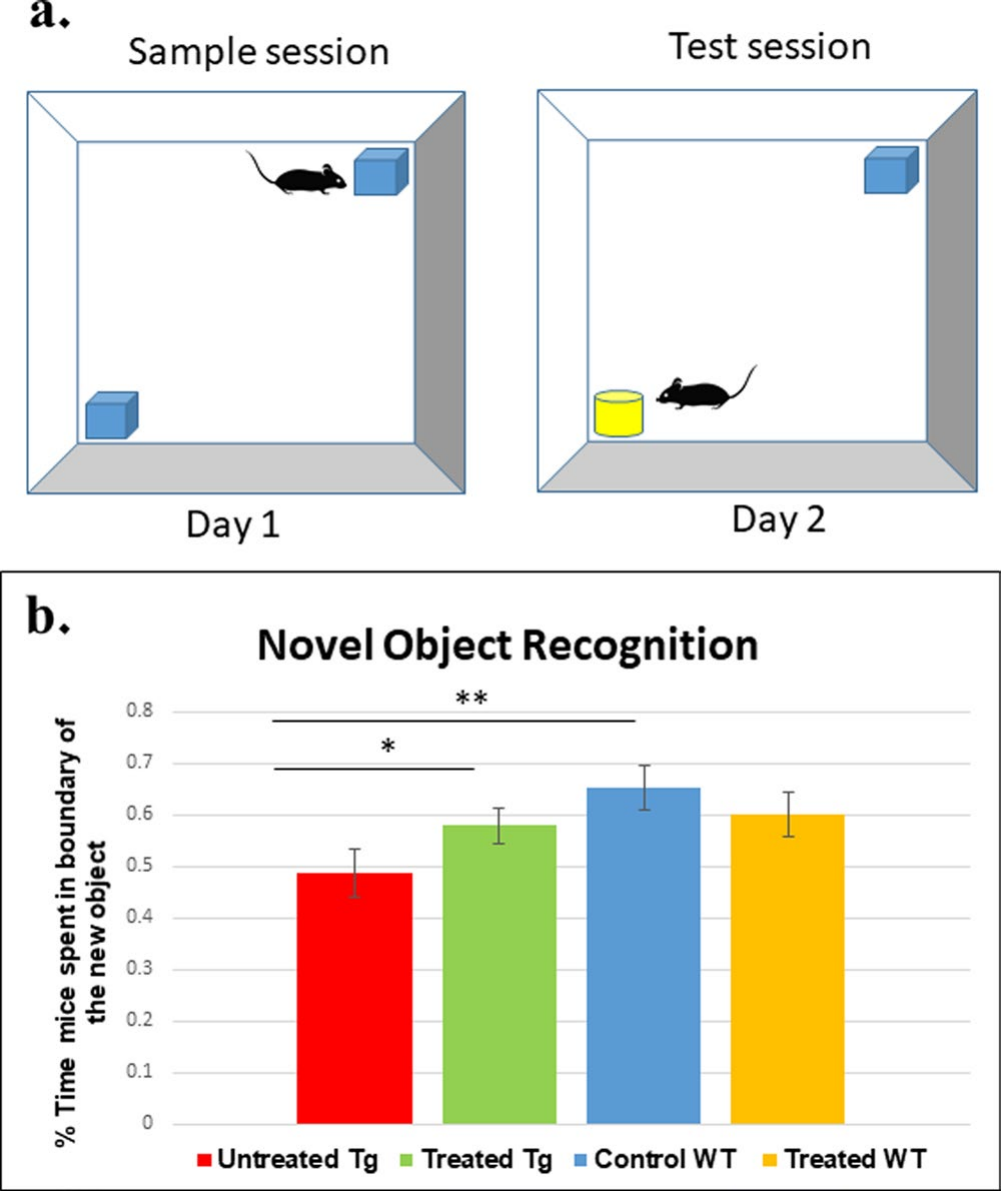
Recognition of a novel object is improved by Nano-PSO treatment of 5XFAD mice. Nano-PSO treated and untreated 5XFAD and WT mice were subjected to the novel object recognition task at 10 months of age as described in the methods. (**a**) Cartoon of the experiment. (**b**) Percentage of time spent in boundary of new object in day 2. Statistical analysis by simple contrast test was performed and showed a trend toward a difference between the groups ([f (3,39) = 0.49 p = 0.077].

Nano-PSO was administrated to 3 weeks old C57BL/6 and 5XFAD mice until they reached 10 months of age. At 7 months of age, groups of treated and untreated mice were subjected to the T-maze (Fig. 2) and the open field habituation tests (Fig. 3). At 10 months of age, the same mice were subjected to the novel object recognition test (Fig. 4).

The T-maze test is used to assess spatial long-term memory impairment^29^. In this test, mice could only enter two arms on day 1, while on day 2 an additional arm was open for exploration (see cartoon in Fig. 2a). WT mice will prefer to explore the new available arm on day 2, while for the 5XFAD mice all the arms are considered as new on day 2. Planned contrasts statistical analysis yielded a significant difference among the groups [f (3,39) = 13.890, p < 0.001)]. As demonstrated in Fig. 2b, quantification of the number of entries to the newly available arm in the second day revealed a significant impairment in the memory of untreated 5XFAD as compared to C57BL/6 mice (P = 0.02). In contrast, the Nano-PSO treated 5XFAD mice performed in this test almost as well as the WT group (P = 0.2). Surprisingly, Nano-PSO treated WT mice presented statistically significant improvement in this memory test as compared to untreated control mice (P = 0.001), suggesting administration of Nano-PSO may prevent age related cognitive decline for both AD and WT mice, at least as measured by this test.

Next, we subjected these groups of mice to the open field habituation task, which is used to evaluate the long-term non-associative spatial memory^30^. Mice were placed in the same exact environment in the first and second days and those with a better memory (WT mice) moved less in the repetitive exam performed in day 2 (Fig. 3a), as opposed to mice that “could not remember” the events of the previous day (5XFAD mice). Results for this test can be seen in Fig. 3b {analyzed by one-way ANOVA: [f (3,40) = 11.290 p < 0.001]}. Each column in Fig. 3b represents the delta between the activity in day 1 to the activity in day 2 for a designated group. There was no significant difference in the locomotion of the mice in day 1 (not shown), indicating differences between the groups in day 2 refer to memory and not to movement abilities. Indeed, the decrease in exploratory behavior following exposure to the already familiar field was significantly higher for the WT mice as compared to the 5XFAD mice, in which the delta was significantly lower (WT vs Tg: p < 0.001), indicating these mice don’t “remember” they have already been in this environment. Contrarily to the untreated group, the Nano-PSO treated 5XFAD mice present a much higher delta between day 1 and day 2 (p = 0.003), indicating that long-term administration of Nano-PSO reduced the time dependent memory deterioration in the AD model mice. No significant difference was observed in this test for the treated WT mice.

A similar trend was noticed in the novel object recognition test performed for all groups at 10 months of age. In this test, mice were subjected in day 1 to two identical objects, while on day 2 one object was replaced by a different one in both color and shape (Fig. 4a). The test measures the preference of individual mice for each of the objects on the second day, when values below 0.5 indicate there is no preference for the new object over the older one^31^. Statistical analysis by simple contrast test was performed and showed a trend toward a difference between the groups ([f (3,39) = 0.49 p = 0.077]. The untreated 5XFAD group (n = 11) achieved an average value of 0.48 (SE = 0.0468). Untreated WT (n = 8) mice prefer the new object on the second day (0.65). In contrast to the untreated 5XFAD mice, the treated Tg group (n = 17) achieved an average value of 0.55 (SE = 0.035) suggesting mice from this group recognize to some degree that there is a new object in the field. There was no effect of Nano-PSO treatment on the object recognition of WT mice (n = 8) (values about 0.6 for treated and untreated Nano-PSO mice). We conclude that long-term administration of Nano-PSO can ameliorate the cognitive deterioration of 5XFAD mice, and in some cases even improve cognition in WT mice, which may point to prevention of normal aging effects.

### Nano-PSO restores COX-IV 1 expression and reduces Aβ accumulation in mitochondria

Mitochondrial deficits are well established in human subjects and animal models suffering from neurodegenerative conditions including AD^2,3^. This may be due to oxidation effects and in the case of AD, also to the accumulation of Aβ in mitochondria^32,33^. Cytochrome oxidase, or complex IV, may be the main enzyme associated with progression of AD, as is the case for other neurodegenerative diseases^34^. Indeed, under oxidative stress and high energy demand, isomer COX IV-1 of complex IV is replaced by COX IV-2, which, as opposed to COX IV-1, can perform its function in an uncoupled form, at least under the levels of ROS became too toxic for cells to survive^35^. We have shown recently that this is also the case for TgMHu2ME199K mice, a model of genetic CJD, and that long term administration of Nano-PSO to these mice restored normal mitochondrial function and expression levels of COX IV-1, even while the levels of disease related PrP were not affected^14^.

**Figure 5:**
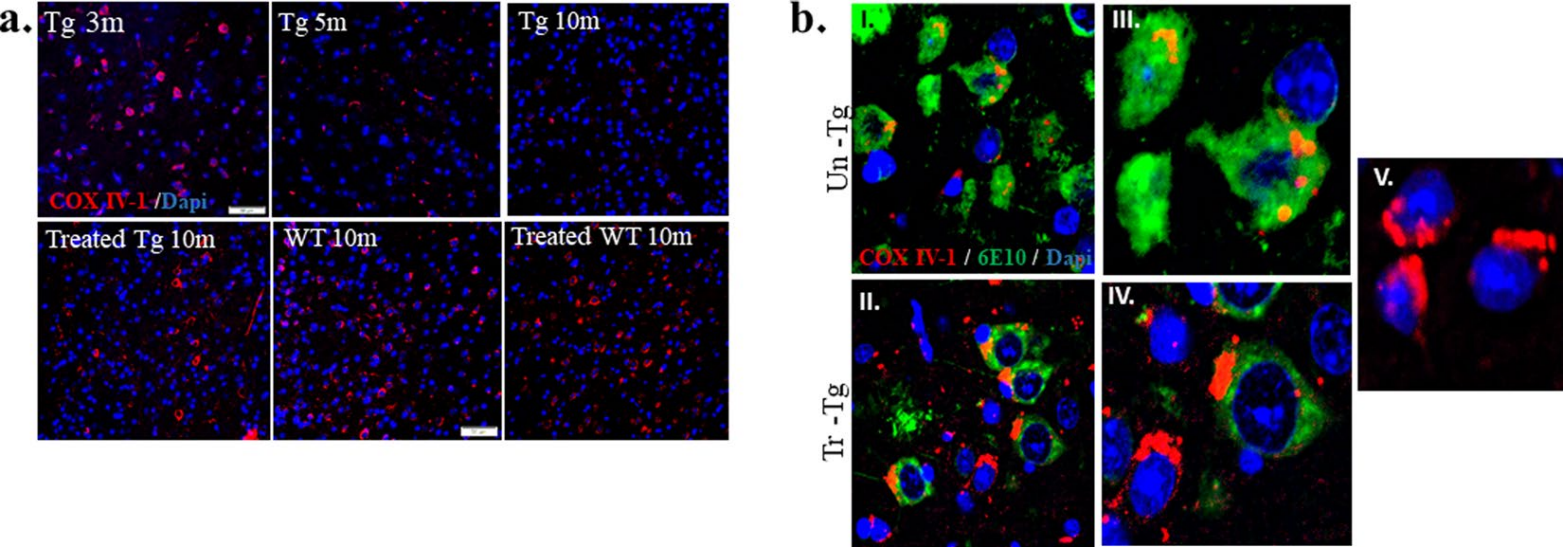
Nano-PSO treatment restored mitochondrial COX IV-1 expression in brains of 5XFAD mice. (**a**) Paraffin embedded brain sections of untreated 5XFAD mice (at 3, 5 and 10 months of age), treated Tg (10 months) as well as untreated and treated WT (10 months) mice were stained with α COX IV-1 mAb (red) and counterstained with dapi (blue). The magnification of cortex area presented in this figure is (x20). (**b**) Ten months old 5XFAD mice, treated (I) and untreated (II) with Nano-PSO, were double-stained with the 6E10 antibody (green) and α COX IV-1 mAb(red) and presented at a magnification of x60. Magnification of single cells (x100) presented for treated (III) and untreated (IV) 5XFAD brains. Cortical neurons from 10 mounts WT brain also stained with 6E10 and COX IV-1, presented in picture V (x100).

To investigate if this is also true for Nano-PSO treated 5XFAD mice, we immunostained brain sections of untreated 5XFAD mice at different ages with an α-COX IV-1 antibody and compared them to brain sections of 10 months old WT mice as well as Nano-PSO treated WT and 5XFAD mice. Figure 5a presents the results of such an experiment. At 3 months of age, when there is still no measurable cognitive loss in the 5XFAD mice, the COX IV-1 staining was similar to the one observed in normal brains, but two months later (5 months old mice) the levels of COX IV-1 in 5XFAD brains were significantly reduced and subsequently there were mostly absent when these mice reached 10 months of age. Contrarily, brain slices of Nano-PSO treated 10 months old 5XFAD mice presented levels of COX IV-1 similar to the ones observed in WT mice. Interestingly, 10 months old treated WT mice present a slight elevation of COXIV-1 expression, concomitant with the improved cognitive performance of such mice in the T-maze test. These results resemble the ones obtained for TgMHu2ME199K mice modeling for genetic CJD^14^.

The 5XFAD mice used in the current study were reported to display age dependent intraneuronal and mitochondrial Aβ aggregates, as well as extracellular plaques comprising Aβ^21^. To investigate the intracellular levels and mitochondrial localization of Aβ in Nano-PSO treated and untreated 5XFAD brains, we co-immunostained 10 months old paraffin embedded brain sections from treated and untreated 5XFAD mice both with the αAβ 6E10 mAb (green) and with *α*COX IV-1 (red), which is an established mitochondrial enzyme. Figure 5b shows an intense staining for Aβ (green) in most cells of the untreated samples. As for the COX IV-1 staining, while in some cells there was no signal whatsoever (as seen in Fig. 5a), in others cells there was an orange signal representing co-localization between Aβ and the mitochondrial protein. Contrarily, in the panel for the Nano-PSO treated brain samples, Aβ levels were significantly lower, and the stronger COX IV-1 signal was mostly red, indicating Aβ levels in mitochondria have been reduced by the treatment. This is an interesting result indicating a significant difference between the effect of Nano-PSO on 5XFAD mice as compared to TgMHu2ME199K mice^28^. While in both models of neurodegeneration COX IV-1 expression was restored by Nano-PSO administration, only in the AD model Nano-PSO reduced the levels of the aberrant protein aggregates.

### Aβ plaques burden is decreased in brain sections of Nano-PSO treated 5XFAD mice

**Figure 6:**
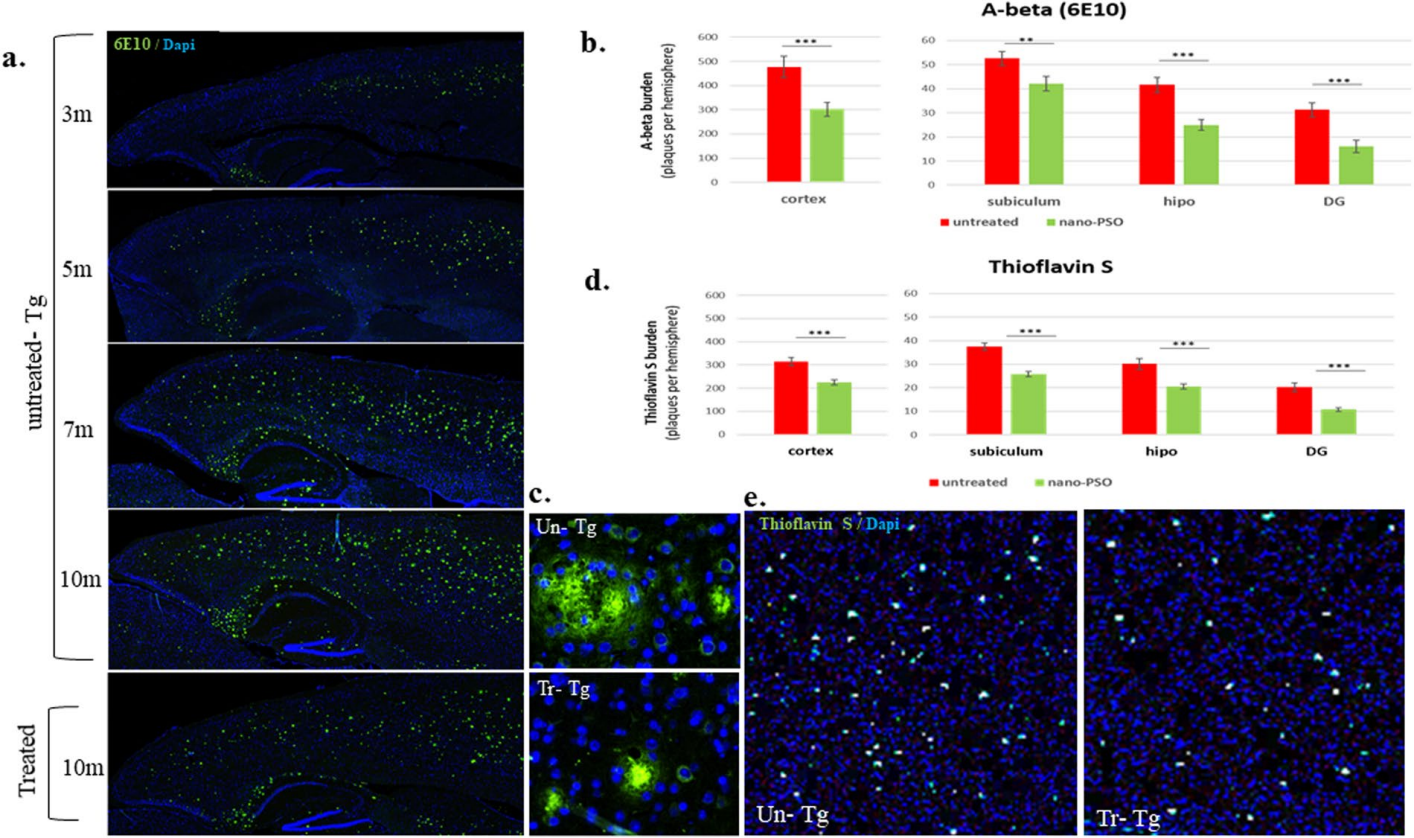
Amyloid plaque burden is decreased in brains of Nano-PSO treated 5XFAD mice. (**a**) Sagittal serial brains sections of - 5XFAD untreated mice in ages of 3, 5, 7 and 10 months and 10 months old Nano-PSO treated mice were immunostained with α-Aβ antibody (green; 6E10 mAb) **(b**) quantitative assessment of Aβ plaques burden per hemisphere of 10 months 5XFAD mice, treated (green) or untreated (red) with Nano-PSO, (four section of each brain; n = 5 per group: unpaired t-test; **p = 0.01; ***p < *0.001*). (**c**) Enlargement of Aβ plaques from 10 m old untreated 5XFAD brain -and Nano-PSO treated 5XFAD brain. **(d**) Quantitative assessment of Thioflavin S dense-core plaques per hemisphere of treated and untreated 10 months 5Xfad mice (four section of each brain; n = 5 per group; unpaired t-test; ***p < 0.001). (**e**) Sagittal section of 10 months 5Xfad mice, treated and untreated, were stain with Thioflavin S, to label dense-core plaques in cortex (x20).

To evaluate whether treatment with Nano-PSO could reduce the amyloid plaque burden in 5XFAD mice we immunostained Aβ plaques in brains of untreated Tg mice of different ages as well as in 10 months old Nano-PSO treated 5XFAD mice both by immunohistochemistry and by thioflavin S^36^. In untreated 5XFAD mice, the levels of Aβ plaques in brains increased in an age-dependent manner^37^ (see Fig. 6a), however this was not the case for the Nano-PSO treated 5XFAD mice. As can be seen in Fig. 6(a–c), there was a significant reduction in Aβ signal strength and number of plaques between treated and untreated mice at 10 months of age. Similar results were obtained by thioflavin S staining (Fig. 6d,e), which measures levels of neuritic plaques in cortex, hippocampus, DG and subiculum^38^.

**Figure 7:**
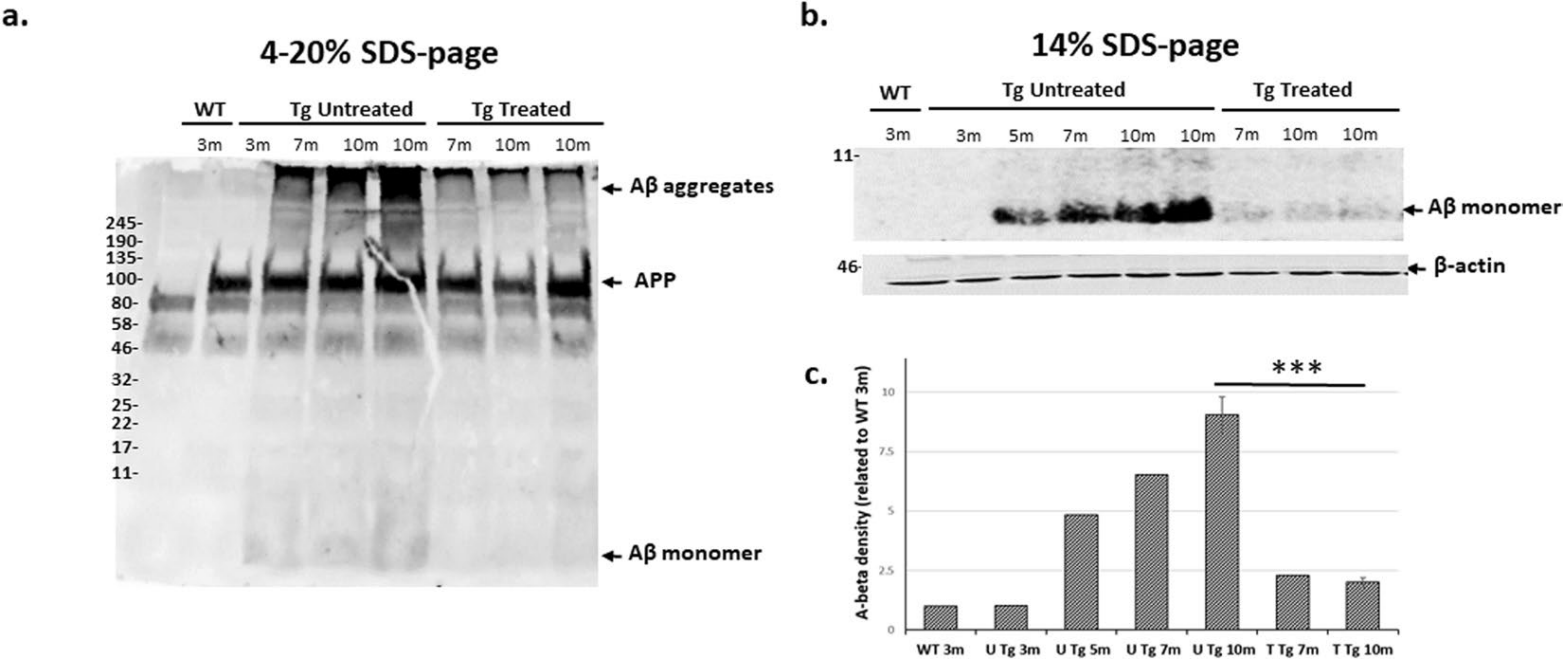
Decreased levels of Aβ in brains of 5XFAD mice treated with Nano-PSO. Brain homogenates of WT, untreated and treated 5XFAD transgenic mice were immunoblotted with the anti Aβ 6E10mAb. Figure **(a)** shows levels of APP and Aβ oligomers. Figure **(b)** shows Aβ monomers in brains. Beta-actin served as loading control (lower panel of b). In some cases, blots have been cropped and increased in exposure; full length original blots are presented in Supplementary Fig. 1. Figure **(c)** represent quantitative analysis of immunoreactive bands. The bars represent the relative levels of Aβ compared with beta actin and are expressed as percentage of the 3m WT value. Unpaired t-test (***p = *0.006*).

Next, we evaluated the levels of APP and Aβ by immunoblotting. APP is a single transmembrane glycoprotein located in most brain regions. Cleavage of APP by beta and gamma secretase yield the A-beta peptide, a toxic fragment which contains 40 or 42 amino acids that can aggregates to form flexible soluble oligomers which may exist in several forms^39^. Brain samples from WT and 5XFAD Nano-PSO treated or untreated mice at several ages were subjected to SDS page by a protocol described in the methods, and subsequently immunoblotted with the αAβ 6E10 antibody, which recognizes human APP as expressed in the 5XFAD transgene, but not mouse APP as expressed in WT mice. Figure 7 presents the results of these experiments. As can be seen in panel a (4–20% acrylamide gradient), while similar APP levels were expressed in all 5XFAD mice, the pathological Aβ band, either in aggregated forms with diverse molecular weight or as a 4 kDa monomer, was detectable only at older Tg mice, already suffering from cognitive impairment. Most important, and concomitant with the reduced number of plaques (as seen in Fig. 6), Aβ levels were significantly reduced in the Nano-PSO treated mice, both at 7 and at 10 months of age. This result can be also observed in panel b, a 14% percent acrylamide gel, which shows better the reduction of the Aβ 4 kDa monomer levels after Nano-PSO treatment. We therefore conclude that long-term administration of Nano-PSO resulted in a significant decrease in both intracellular and extracellular Aβ levels.

### Reduction of p25 levels in the brains of Nano-PSO treated mice

We have shown above that following Nano-PSO administration, CLA, an 18:2 conjugated fatty acid recognized as a calpain inhibitor^20^, accumulates in the brains of Nano-PSO treated mice. This is a unique effect which does not occur following administration of PSO or CLA in their native forms to animals^17–19^. Inhibition of calpain activity is acknowledged today as a potential target in the search of treatments for neurodegenerative diseases since high activity of calpain and the subsequent accumulation of its product, p25, is a feature in diseases such as AD and PD^8,24^. Brain accumulation of p25 was also shown in the 5XFAD model^21^.

**Figure 8:**
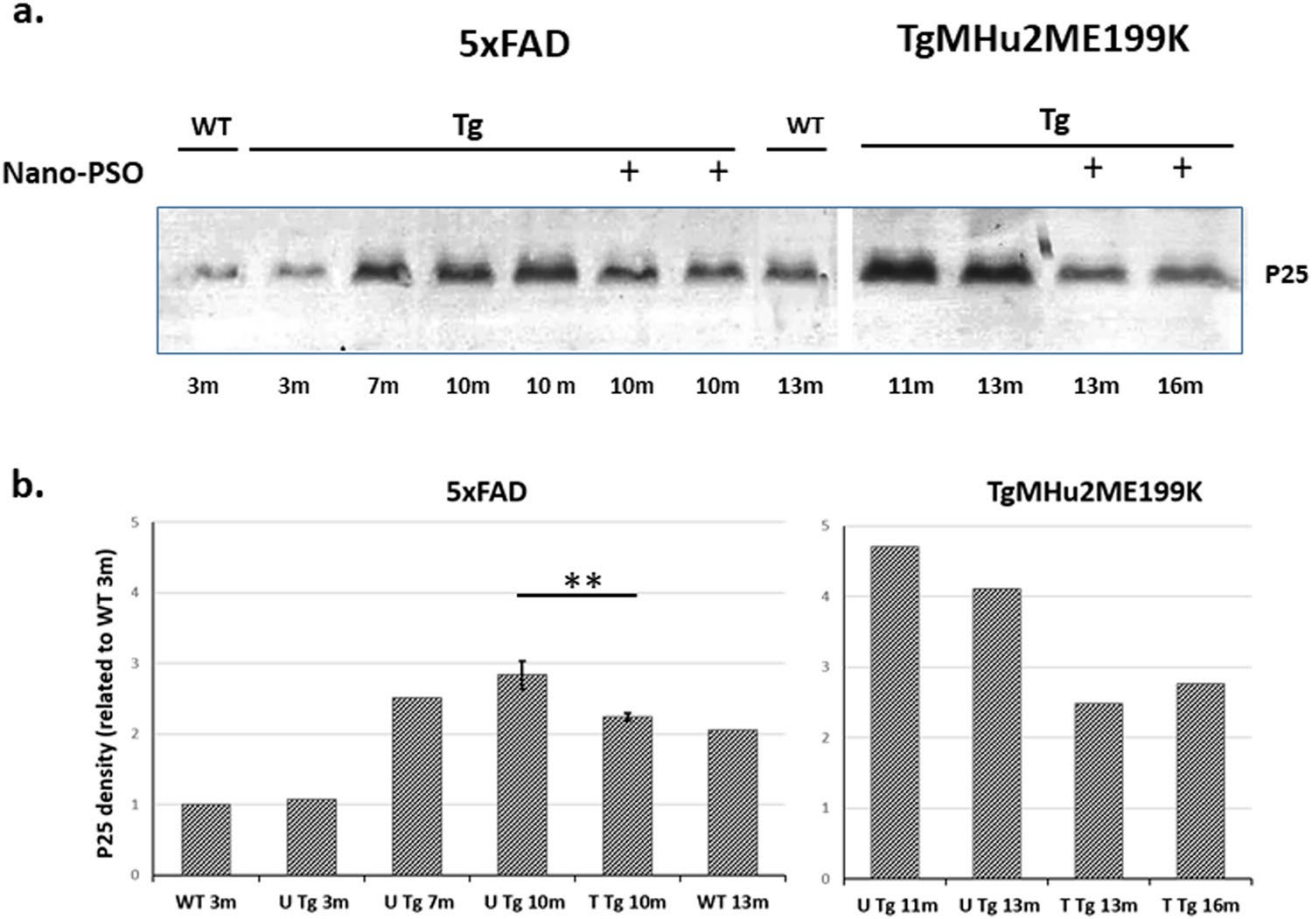
Nano-PSO treatment reduced p25 levels in neurodegenerative brains. (**a)** Brain homogenates of 5XFAD and TgMHu2ME199K mice treated and untreated with Nano-PSO as well as brain homogenates from WT mice of different ages were immunoblotted with a p35/ p25 antibody. Blots have been cropped for conciseness; full length original blots are presented in Supplementary Fig. 2. **(b)** Quantitative analysis of immunoreactive bands of p25. The bars are expressed as percentage of the 3 m WT value. Unpaired t*-test* revealed significant difference between the treated and untreated 5XFAD mice (**p < *0.05*) and treated and untreated TgMHu2ME199K mice (*p* < *0.02*).

To test the levels of p25 in the Nano-PSO treated and untreated mice, brain homogenates from 5XFAD mice of different ages representing subclinical or clinical stages of AD as well as of WT C57BL/6 mice were immunoblotted with an α p35-p25 antibody. We also immunoblotted with the same antibody brain sampled from Nano-PSO treated and untreated TGMHu2ME199K mice^13,15^, to establish whether p25 can also serve as a marker for neurodegeneration and treatment success in genetic prion diseases. Figure 8 shows that the levels of p25 are low in young WT mice and 5XFAD mice, then increase significantly in the clinical stages of both 5XFAD and TgMHu2ME199K mice. In both animal models, mimicking AD and gCJD respectively^13,21^, p25 was reduced following long-term Nano-PSO treatment, concomitantly with clinical improvement and reduction of oxidative stress damage.

## Discussion

We have shown in this work that administration of Nano-PSO, a Nano-formulation of PSO previously shown to delay disease progression in a genetic model of CJD as well as in EAE^40^, resulted in incorporation of substantial levels of CLA into the brains of treated mice. These results are in contrast with those obtained for animals treated with PSO, following which, as previously described^17,19^, no PA and only traces of CLA were detected in brains of treated animals. CLA, a metabolite of PA, was also absent from the brains of mice treated with other oils comprising Punicic Acid and even form brains of animals treated directly with CLA^17,18,41^. Targeting CLA to the brain may be of distinct importance for the treatment/prevention of neurodegenerative diseases due to its classification as a calpain inhibitor^20,42–44^. As such, CLA in cell culture demonstrated anti Aβ features and reduces p25 accumulation^20^. In this work, we have shown that long-term administration of Nano-PSO exerts a profound beneficial effect on 5XFAD mice^45^, significantly reducing their age-dependent cognitive decline.

To establish whether the mechanism of action of Nano-PSO in the AD model indeed results from inhibition of calpain activity, we looked into the levels of p25, the calpain product which binding to CDK5 results in deregulation of this important enzyme, eventually leading to neurodegeneration^46–48^. As can be seen in Fig. 8, while p25 accumulates in the brains of mature and older 5XFAD mice, its levels were significantly reduced in the brains of same age 5XFAD mice to which Nano-PSO was administrated continuously since young age. Interestingly, this was also true for the p25 levels in the brains of TgMHu2ME199K mice. This fact is particularly surprising since in these mice, while Nano-PSO exerts an intense beneficial clinical effect, it does not induce any reduction in the age depended accumulation of disease related PrP^28^, the mayor feature of prion diseases such as genetic CJD^49^. Calpain inhibitors were also shown to reduce p25 in Parkinson’s disease models as well as in Huntington disease^10,50^.

Most interestingly, p25, as opposed to the normal CDK5 activator (p35), is not readily degradable and tends to accumulate in brain of subjects suffering from neurodegenerative diseases^24^. This metabolic stability may be the cause for the deregulated activity of CDK5, eventually leading to neurodegeneration. P25 may therefore be added to the list of aberrantly folded and non-degradable peptides recognized as prion like proteins in individual neurodegenerative diseases^51^, and even be considered as a “universal prion”, a stable metabolic central regulatory protein which accumulation and subsequent aberrant activity vis a vis CDK5 causes brain damage in all neurodegenerative diseases. Whether the accumulation of p25 is the result or the cause of individual prion and prion like proteins accumulation remains to be established. The fact that long-term Nano-PSO administration decreases the levels of p25 in both AD and CJD animal models, concomitant with delay of clinical disease advance, reinforces the notion that brain targeted calpain inhibitors, in particular safe reagents that can be administered for long term periods, may be used as preventive treatments for an array of brain diseases.

## Methods

### Generation of drinking water containing Nano-PSO

Generation of self-emulsifying Nano-PSO is described in patent no. 14/523,408. For drinking water preparation, 16.5 ml of Nano-PSO self-emulsion formulation was diluted into 300 ml of water to form a white emulsion with a final concentration of 1.6% oil. Mice were allowed to drink freely from the diluted formula.

### Mice

We used 5XFAD mice^21,45,52^ grown in our animal facility. The mice were crossed with C57BL/6 mice and their offspring screened to identify the presence of app/ps-1 transgenes. Mice comprising both transgenes were used for the experiments, while the non-Tg mice served as WT mice. 5XFAD mice developed amyloid plaques from around 2 months of age and cognitive deficits from 4–5 months of age^21^. All animal experiments were conducted under the guidelines and supervision of the Hebrew University Ethical Committee, which approved the methods employed in this project (Permit Number: MD-17-15226-2).

### Treatment of 5XFAD mice

Nano-PSO was administered to 3 weeks old mice of experimental groups by adding the self- emulsion formulation to their drinking water [treated Tg n = 18 (7 males and 11 females); treated WT n = 8 (2 males and 6 females), untreated Tg n = 12 (4 males and 9 females); untreated WT n = 8 (2 males and 8 females)]. It was previously shown that there are no significant differences in cognitive performance tests between male and females in this transgenic line^53^. Water containers of all groups were replaced twice a week. Mice were sacrificed several days after the completion of the cognitive test performed at 10 months of age and their brains collected and processed for immunohistochemistry and immunoblotting experiments.

### Behavioral tests

The following cognitive tests were performed on two groups of either 5XFAD or C57B mice, at either 7 or 10 months of age. The results presented in this manuscript represent the average of the two groups. Cognitive experiments were performed in the facilities of “Hadassah BrainLabs - National Knowledge Center for Research in Brain Disorders”.

### T-maze

The T maze test was use for assessing the spatial long-term memory, measuring exploratory behavior in animals, as described in Benharmon at all^54^. Shortly, the maze contains 2 arms of 45 cm length and 10 cm width that extended at a right angle from a 57-cm-long alley. The test comprises two trails with an interval of 24 hours. On the first day mice are placed in the start arm of the maze, and allowed to explore it for 8 minutes, while one of the short arms is closed. On the second day, both arms are open, and the animal is allowed to explore all maze parts for 3 minutes. The number of entries to the unfamiliar arm and the time spent there were recorded. Normal healthy animals prefer to visit the new arm of the maze on the second day rather than the familiar arm.

### Novel object recognition

The novel object recognition test is used to evaluate cognition, especially non-spatial recognition memory. On the training day, the animals were placed in a 25 × 25 cm arena containing two identical objects for 10 minutes and then returned to their home cages. On the testing day, 24 hours later, the mice placed in the same open arena, with one familiar object and one new object, different in shape, color, and texture from the familiar one. Each mouse was allowed to explore the arena for 4 minutes, while the time it spent exploring the new or old object were recorded by the sniffing activity around the new object. The ratio of exploration of the novel object and the total exploration of the two objects were calculated and presented in the figure. The test was performed using the Ethovision 10 system, providing fully computerized, blinded and unbiased measurement. Normal animals tent to explore the new object longer than the old one, indicating normally long-term recognition memory^55^.

### Open field habituation task

The open-field habituation test evaluates long-term non-associative spatial memory and learning, by measuring the decrease in the exploratory activity of the animal in a test session carried out 24 h after the first (exploration) session^56,57^. On the training day, animals were exposed to a novel environment: placed in a 40 cm × 50 cm × 60 cm open field box for a 5-min period. Twenty-four hours later, animals were re-exposed to the same environment. Locomotion on the training and testing days were recorded using the Ethovision 10 system, providing fully computerized, blinded and unbiased measurement. Bigger delta between days (shorter distance on the test session compared to the training session) represented intact learning^30^.

### Pharmacokinetic studies

For detection of PA and CLA in brains and livers after one single dose administration, 50 µl PSO or 170 µl Nano-PSO (containing 50 µl PSO) were intragasticaly administered to C57BL/6 mice (n = 3 per group) and sacrificed four hours thereafter, together with two untreated mice. For detection of PA and CLA following continues administration, the same daily dose of PSO was administered intragasticaly to C57BL/6 mice (n = 6) for two weeks. In the Nano-PSO group (n = 6), the self-emulsion formulation was added to their drinking water at the same equivalent dose of PA (daily dose of 50 µl PSO per mouse). Mice were sacrificed under anesthesia two hours after the administration of last dose. Liver and brain tissues were quickly collected and stored at −80 °C until analysis.

### Analysis of lipid content

Frozen brain and liver samples collected in the pharmacokinetic experiments were sent to the Milouda-Migal laboratories, Milu’ot South Industrial Zone, Israel.

#### Lipid extraction from animal tissue

Samples containing 100–200 mg of tissue were weighted into a distillation flask. 2 ml of internal standard solution Methyl tridecanoate (purity ≥ 97%), 100 mg of Pyrogallol and 100 ml of HCL (4 M) were then added to the sample and were stirred thoroughly. The solution was then placed for 60 min at boiling temperature and was mixed every 10 min. 100 ml of hot water and Celite were then added and the solution was filtered using Whatman filtration paper. The filter paper was then rinsed with hot water until the filtrate was neutralized. The filter paper was then placed in a beaker which was put into an oven at 50 °C for overnight dehydration following extraction by Soxtec 2050 (Tecator). The oil extracted was then weighted and held at −20 °C.

#### Quantification of CLA and PA

Previously extracted oil was placed in a distillation flask and 0.5 M NaOH in Methanol was added. The solution was stirred and placed for a 10 min reflex. When the solution had reached boiling, BF_3_ and Isooctane were added. After cooling, 20 ml of saturated NaCl solution was added and vigorously vortexes. The supernatant containing the fatty acid methyl ester (FAME) was collected and quantified by gas chromatograph, GC.

#### Gas chromatograph analysis

Analysis was performed on Agilent Technologies 6890 N network GC system, CA, USA equipped with a capillary injection system, split mode ration of approximately 1:100, SP^TM^ – 2560 fused silica capillary column (100 m × 0.25 mm with 0.20 μm film thickness, Supelco inc. Bellefonete, PA, USA) and flame ionization detector (FID). The oven temperature programing was isothermal at 170 °C. The helium carrier gas flow rate was 19 cm/sec. Injection temperature was 250 °C and detection temperature was 250 °C. Peaks were identified using Openlab software.

### Immunohistochemistry studies

Histological evaluations were performed on 5 µm paraffin-embedded sections of brain samples. Sections were stained for mouse α-human Aβ, clone α-6E10 and Thioflavin S (sigma Aldrich) to asses amyloid plaques, and with α COX IV-1 rabbit monoclonal antibody raised against a human COX IV-1 peptide which in mice recognizes only the COX IV-1 isomer (for antibodies details see Supplementary Table 1). Secondary antibodies coupled to Alexa Fluor 488 and 568 were purchased from Abcam. Nuclei were labeled with DAPI Fluoromount (Vector Laboratories). Confocal analysis was performed by a Nikon A1R Confocal Laser Microscope System using the NIS-Elements C control software.

### Western blotting

Brain extracts from 5XFAD transgenic mice were homogenized at 10% (W/V) in 10 mM Tris–HCl, pH 7.4 and 0.3 M sucrose. Samples normalized by pierce BCA protein assay kit (ThermoFisher scientific, USA) to 80 µg proteins in each brain sample for WB detection. Samples were subsequently boiled in the presence of urea and SDS, subjected to 14% SDS PAGE or 4–20% gradient SDS PAGE (Bio-Rad) and transferred to nitrocellulose membrane for 1.5 h, 300 mA. Membranes were then treated as described previously^45^. Shortly, membranes were transferred to PBS, and epitopes retrieved by boiling the membranes twice for 10 sec each time, with a 4 min cooling period after each episode of boiling. After blocking with 3% milk for 1 h, the boiled membranes were immunoblotted either with 6E10, or with α p35/p25 mAb. The 6E10 membrane was re-probed with α β-actin to examine the levels of total protein loaded onto the membrane. Immunoreactive bands were analyzed using the ImageJ software. For antibodies details see Supplementary Table 1.

### Statistical studies

The data of the behavioral tests are presented as mean ± standard error of the mean (S.E.M). Statistical analysis was performed using IBM SPSS Statistics V.23. Data was analyzed using either one-way ANOVA for OFH task and by contrast analysis for the T-maze and novel object recognition tests. Statistical analysis for additional experiments (pharmacokinetics, quantification of immunoblots and immunohistochemistry) was done by t-test analysis.

### Ethics approval and consent to participate

These experiments were conducted under the guidelines and supervision of the Hebrew University Ethical Committee, which approved the methods employed in this project (Permit Number: MD-17-15226-2).

## Supplementary information

Supplementary Material
