# Author Correction: Brain targeting of 9c,11t-Conjugated Linoleic Acid, a natural calpain inhibitor, preserves memory and reduces Aβ and P25 accumulation in 5XFAD mice

**DOI:** 10.1038/s41598-020-58232-y

**Published:** 2020-01-23

**Authors:** Orli Binyamin, Keren Nitzan, Kati Frid, Yael Ungar, Hanna Rosenmann, Ruth Gabizon

**Affiliations:** 1 Department of Neurology, The Agnes Ginges Center for Human Neurogenetics, Hadassah-Hebrew University Medical Center, Jerusalem, Israel; 2 Chemistry laboratory, Milouda & Migal Laboratories, Merieux Nutrisciences, Milu’ot South Industrial Zone, Akko, Israel

Correction to: *Scientific Reports* 10.1038/s41598-019-54971-9, published online 05 December 2019

The original version of this Article contained an error in Affiliation 2, which was incorrectly given as ‘Chemistry laboratory, Milouda & Migal Laboratories, Meriux Nutrisciences, Milu’ot South Industrial Zone, Akko, Israel’. The correct affiliation is listed below:

Chemistry laboratory, Milouda & Migal Laboratories, Merieux Nutrisciences, Milu’ot South Industrial Zone, Akko, Israel.

In addition, the Author Contributions section in the original version of this Article was incorrect.

“O.B. performed experiments, treated the mice and performed immunohistochemistry and western blot studies and edited the manuscript. K.N. performed the Behavioral tests and relevant statistics. K.F. assisted when needed. Y.U. performed the pharmacokinetic studies. H.R. donated the 5XFAD line and counsel about the experiments. R.G. conceived experiments and wrote the manuscript. All authors read and approved the final manuscript.”

now reads:

“O.B. performed experiments, treated the mice and performed immunohistochemistry and western blot studies and edited the manuscript. K.N. performed the Behavioral tests and relevant statistics. K.F. assisted when needed. Y.U. performed the pharmacokinetic studies. H.R. counsel about the behavioral experiments. R.G. conceived experiments and wrote the manuscript. All authors read and approved the final manuscript.”

These errors have now been corrected in the HTML and PDF versions of the Article.

