# Supplementary material for: Brain targeting of 9c,11t-Conjugated Linoleic Acid, a natural calpain inhibitor, preserves memory and reduces Aβ and P25 accumulation in 5XFAD mice
Source: Sci Rep. 2019 Dec 5;9:18437. doi: 10.1038/s41598-019-54971-9 (PMC6895090; doi:10.1038/s41598-019-54971-9)
Supplement: Supplementary file 1 — Supplementary Material [file 41598_2019_54971_MOESM1_ESM.docx]

**Brain targeting of 9c, 11t Conjugated Linoleic Acid, a natural calpain inhibitor, preserves memory and reduces Aβ and P25 accumulation in 5XFAD mice**

**Supplementary Material**

**Orli Binyamin^1^, Keren Nitzan^1^, Kati Frid1, Yael Ungar^2^, Hanna Rosenmann^1^ and Ruth Gabizon^1^**

1: Department of Neurology, The Agnes Ginges Center for Human Neurogenetics, Hadassah-Hebrew University Medical Center, Jerusalem, Israel

2: Chemistry laboratory, Milouda & Migal Laboratories , Meriux Nutrisciences, Milu'ot South Industrial Zone, Israel

**Supplementary Table 1:** Immunostain and WB antibodies

| Protein Target | Species Raised in; Monoclonal or Polyclonal | Dilution | Manufacturer and Catalog Number |
| --- | --- | --- | --- |
| COX IV-1 | rabbit monoclonal | 1:1000 IF | Abcam, ab202554 |
| 6E10 (anti β-Amyloid 1-16) | Mouse monoclonal | 1:750 IF 1:1000 WB | BioLegend, SIG-39320 |
| Β-actin | Mouse monoclonal | 1:25,000 WB | Abcam, ab49900 |
| P35/p25 | Mouse monoclonal | 1:1000 WB | Santa Cruz, sc-518009 |

**Supplementary Figure 1:** Full-length original western blots for 5xFAD brains with 6E10 antibody (a) and β actin (b) on the same membrane.

**WT**

**Tg Untreated**

**Tg** **Treated**

3m 5m 7m 10m 10m

3m

7m 10m 10m

a.


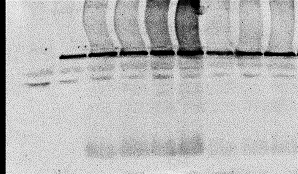


11 kb

Aβ monomer





b.

46 kb

**Supplementary Figure 2:** Full-length original western blots for 5xFAD and TgMHu2ME199K brains with p25/35 antibody.


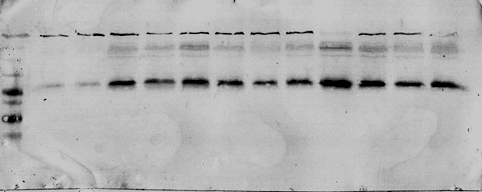


25 kb

**11m 13m 13m 16m**

**13m**

**3m 7m 10m 10 m 10m 10m**

**3m**

**5xFAD**

**TgMHu2ME199K**

**Tg**

**WT**

+ +

+ +

**WT**

**Tg**

**Nano-PSO**
